# Rational Design of Flexible Mechanical Force Sensors for Healthcare and Diagnosis

**DOI:** 10.3390/ma17010123

**Published:** 2023-12-26

**Authors:** Hang Zhang, Yihui Zhang

**Affiliations:** 1 School of Materials Science and Engineering, Nanyang Technological University, 50 Nanyang Avenue, Singapore 639798, Singapore; hang.zhangh@ntu.edu.sg; 2 Applied Mechanics Laboratory, Department of Engineering Mechanics, Tsinghua University, Beijing 100084, China

**Keywords:** mechanical force sensor, flexible structure, healthcare, medical diagnosis

## Abstract

Over the past decade, there has been a significant surge in interest in flexible mechanical force sensing devices and systems. Tremendous efforts have been devoted to the development of flexible mechanical force sensors for daily healthcare and medical diagnosis, driven by the increasing demand for wearable/portable devices in long-term healthcare and precision medicine. In this review, we summarize recent advances in diverse categories of flexible mechanical force sensors, covering piezoresistive, capacitive, piezoelectric, triboelectric, magnetoelastic, and other force sensors. This review focuses on their working principles, design strategies and applications in healthcare and diagnosis, with an emphasis on the interplay among the sensor architecture, performance, and application scenario. Finally, we provide perspectives on the remaining challenges and opportunities in this field, with particular discussions on problem-driven force sensor designs, as well as developments of novel sensor architectures and intelligent mechanical force sensing systems.

## 1. Introduction

The proportion of the world’s population over 60 years old is estimated to nearly double from 12% to 22% between 2015 and 2050 [1], according to World Health Organization (WHO). The demand for personalized, long-term care (LTC) is expected to accelerate with the increase in age. Preventive medical approaches can detect and predict diseases in their early stages, while digitized health indicators can assist doctors in achieving a precise diagnosis, together increasing the cure rate, reducing treatment costs, and improving the quality of life. As a critical part of the real-time health monitoring and digitalized diagnosis, wearable/portable biosensors are of increasing interest in both academia and relevant industries [2,3,4,5,6,7,8,9,10,11,12,13,14,15,16,17,18,19,20,21,22,23,24,25,26,27,28,29,30,31,32,33,34,35,36]. Within the human body, routine physiological processes produce a variety of mechanical forces, such as intracranial pressure (ICP), intraocular pressure (IOP), and blood pressure, all of which serve as critical health indicators. Traditional hard mechanical force sensors (e.g., silicon pressure sensor), primarily rely on the piezoresistive effect, and have been extensively investigated in recent decades [37,38,39,40,41,42,43,44,45,46,47,48,49,50,51,52,53,54]. These force sensors have been widely used in the automotive industry, aerospace engineering, and medical instruments, due to their excellent performance and ease of mass production. For example, Xu et al. utilized the peninsula–island structure to develop a novel micro-electromechanical system (MEMS) piezoresistive pressure sensor with high sensitivity (0.06 mV V^–1^ Pa^–1^) for ultralow-pressure measurement (0–500 Pa) [38]. The trade-off between high sensitivity and linearity has been alleviated, resulting in a MEMS sensor with only 0.36% nonlinearity relative to the full scale. Mikhail et al. further developed a mathematical model for a highly sensitive piezoresistive sensor with a range from −0.5 to 0.5 kPa [39]. The relationship between sensitivity (34.5 mV kPa^−1^ V^−1^) and nonlinearity (0.81% full scale) can be established through the optimization of the silicon membrane and rigid islands geometry. However, traditional hard mechanical force sensors, typically constructed with rigid structures or materials, face challenges in conforming to curved or uneven surfaces, thereby setting limitations to their applications in wearable biosensors. In comparison, flexible mechanical force sensors are capable of accurately detecting and quantifying a wide range of such forces, when attached to the tissues and organs of the human body. When integrated with a customized data collection board and signal analysis algorithms, these flexible force sensors can seamlessly collect and transmit signals [55,56,57,58]. These prominent capabilities of flexible mechanical force sensors enable promising applications in daily healthcare and medical diagnosis [55,59,60,61,62,63,64,65,66,67,68,69,70,71,72,73].

**Figure 1:**
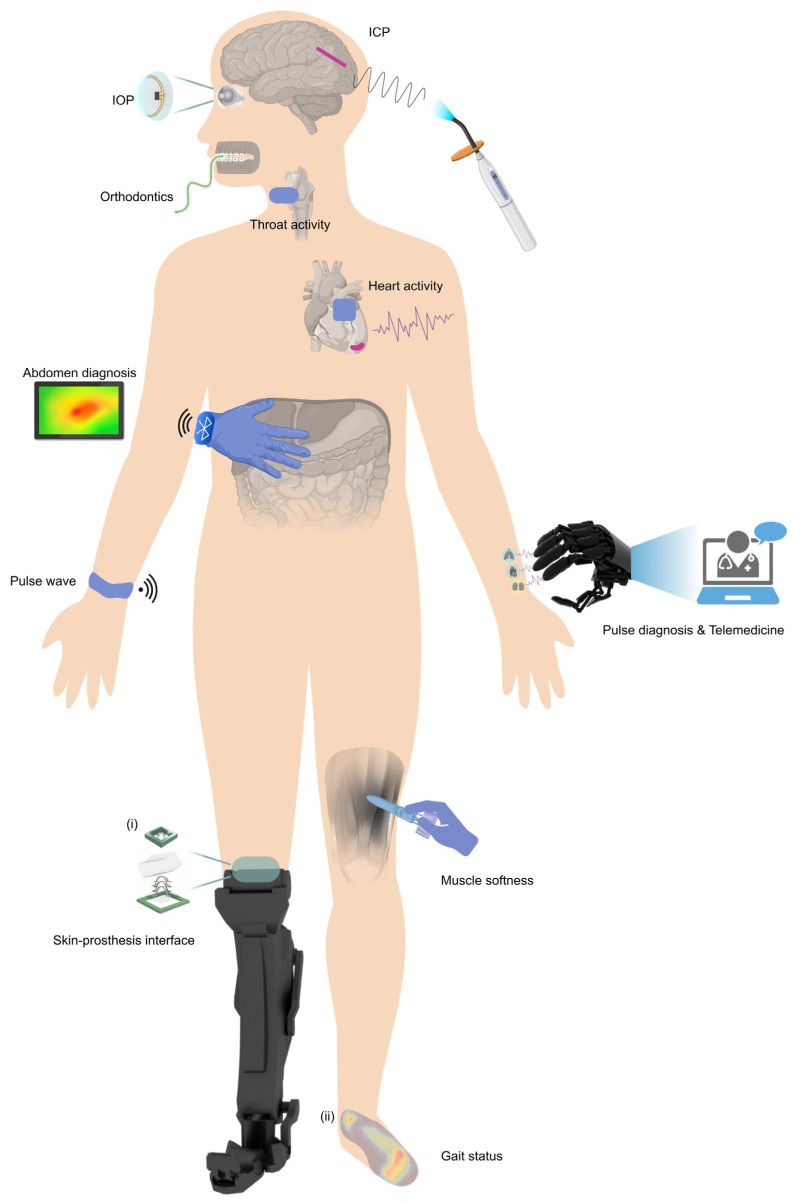
Representative progress in flexible mechanical force sensors for potential applications in daily healthcare and clinical diagnosis, such as pulse wave and muscle softness detection, ICP and IOP measurement, gait status monitoring, palpation, and pulse diagnoses, among others. Panel (**i**) is adapted with permission from Ref. [64]. Copyright 2020, American Association, for the Advancement of Science. Panel (**ii**) is adapted with permission from Ref. [123]. Copyright 2020, Wiley-VCH.

The design of flexible mechanical force sensor usually relies on a strategically designed flexible structure integrated with advanced functional materials [74,75,76,77,78,79,80,81,82,83,84,85,86,87,88,89,90,91,92,93,94,95,96,97,98,99,100,101,102,103]. To improve the biological compatibility of the device with human body, numerous biomaterials are developed for the application of a wide range of medical fields covering orthopedics, drug administration, dentistry, skin tissue engineering, and cardiovascular systems [104,105,106]. These biomaterials usually are non-toxic and designed to closely mimic the mechanical properties of human tissues. Through various working mechanisms (e.g., piezoresistive effect, capacitive effect, piezoelectric effect, triboelectric effect, among others), the force applied to the sensor can be converted into electrical signals. Recent studies have demonstrated that flexible mechanical force sensors with specific architectures can provide outstanding sensing performance in terms of one or multiple aspects. For example, Cai et al. strategically positioned spatially distributed microprotrusions onto a patterned metal film (PMF) [78]. These protrusions switched the minimal out-of-plane compression of the PMF to a significant bending deformation, resulting in a remarkable increase in the sensitivity of pressure measurement by over 177 times. The 3D capacitive force sensor fabricated by the controlled buckling-guided assembly [107,108,109] could achieve high-precision force sensing (resolution ~5.22 nN) and a broad range of tunable sensitivity (by ~33 times), simultaneously [75]. These methods enrich the design and optimization strategies for flexible mechanical force sensors, enabling the customization of sensor performance based on specific application scenarios. Recent advancements in flexible mechanical force sensors have shown promising developments by increasing sensitivity, achieving tunable sensitivity, and improving linearity. These advancements have demonstrated significant applications in various fields, including consumer electronics, robotics and automation, and biomedical monitoring. However, it remains challenging to improve the sensors performance from multiple aspects simultaneously and realize the on-demand design of mechanical force sensors for specific application scenarios. As shown in Figure 1, many applications in healthcare and diagnosis are opened up by developments of flexible mechanical force sensors, including pulse wave [110,111,112,113,114,115] and muscle softness detection [116], ICP and IOP measurement [117,118,119], throat and cardiac activity monitoring [55,120], abdomen and pulse diagnoses [121,122], as well as force sensing in orthotics [123,124], orthodontics [125] and skin-prosthesis interface [64]. While many excellent reviews of flexible pressure sensors can be found in the literature [126,127,128,129,130,131,132,133,134,135,136,137,138,139,140,141,142], they focus mainly on the working mechanisms, material selections and device performances. A comprehensive review that discusses the evolution of their structure and summarizes the interplay among the sensor architecture, performance, and biomedical applications is lacking.

**Figure 2:**
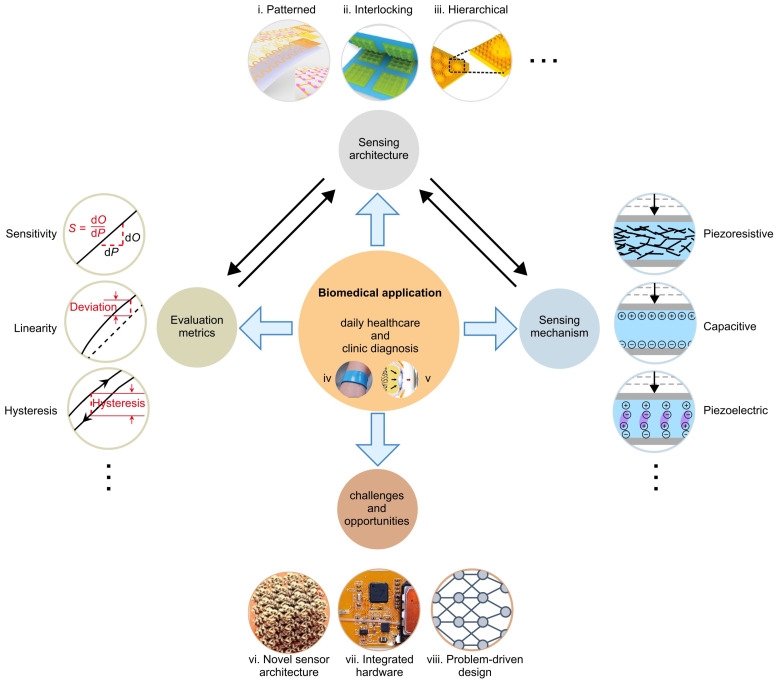
Illustration of the structure of this review. Panel (**i**) is adapted with permission from Ref. [78]. Copyright 2021, American Association for the Advancement of Science. Panel (**ii**) is adapted with permission from Ref. [143]. Copyright 2020, Wiley-VCH. Panel (**iii**) is adapted with permission from Ref. [144]. Copyright 2019, American Chemical Society. Panel (**iv**,**vii**) are adapted with permission from Ref. [56]. Copyright 2023, Springer Nature. Panel (**v**) is adapted with permission from Ref. [145]. Copyright 2017, Springer Nature. Panel (**vi**) is adapted with permission from Ref. [74]. Copyright 2020, Springer Nature. Panel (**viii**) is adapted with permission from Ref. [146]. Copyright 2023, Springer Nature.

In this review, we summarize recent advances in flexible mechanical force sensors, highlighting their wide-ranging applications in daily healthcare and medical diagnosis, and provide perspectives on remaining challenges and opportunities in this field (Figure 2). Section 2 presents an overview of six representative types of flexible force sensors based on different working mechanisms, and twelve representative types of metrics used for measuring the performance of force sensors. Section 3 and Section 4 focus on the representative progress of each type of flexible mechanical force sensors from the perspective of sensor architecture and their biomedical applications. Lastly, we provide perspectives on the existing challenges and open opportunities in the future development of flexible mechanical force sensors for healthcare and diagnosis.

## 2. Fundamentals of Mechanical Force Sensors

The practical application scenarios usually pose specific requirements for the configuration, deformability, and sensing performance of force sensors. For instance, in industrial automation, we often need sensors with robust structures that can survive harsh environments and high loadings. These sensors must also be capable of detecting subtle changes in force or pressure to ensure the smooth operation of machinery. Conversely, in healthcare and diagnosis, the demand usually involves the flexibility and non-invasiveness of sensors, which allows them to conform to the time-dynamic, curvy surfaces of biological organs without inducing large mechanical constraints [111,145,147,148,149,150,151,152,153,154,155,156,157]. The typical pressures resulting from normal touch and human body circulation primarily fall below 100 kPa [61,158]; therefore, the force sensors need to possess a high level of sensitivity in order to capture vital physiological data accurately, without causing discomfort to the patient.

Even within the human body, the characteristics of force signals generated by physiological activities can vary significantly. Specifically, the pressure range for intra-body pressures (e.g., ICP and IOP) typically falls between 1 and 10 kPa, while the pressure range for wearable blood and pulse monitoring devices is generally in the range of 10–100 kPa [158,159,160,161]. Apart from the dynamic force measurement, static force sensors with high precision are also extensively used in assessing muscle softness and conducting abdominal examinations [116,121]. Moreover, different regions of the human body demand distinct geometric shapes and deformation capabilities of force sensors. For instance, a flat film may be employed beneath the foot to track gait status [123,162], whereas a spherical sensor is necessary for monitoring IOP [117]. The mechanical properties of flexible mechanical force sensors are also very important for wearable electronics. For example, a flexible mechanical force sensor should exhibit low bending stiffness for conformal contact, possess biomimetic J-shaped stress–strain curves to minimize strain mismatch with tissues, and demonstrate sufficient fracture toughness for prolonged usage. Note that the performance of the mechanical force sensors can be affected by the operating environment, particularly temperature. Fluctuations in environmental temperature may result in the expansion or contraction of component materials, leading to drift in the calibrated force sensor and affecting sensitivity, linearity, and other performance metrics. During seasons such as summer and winter, the temperature variance between indoor and outdoor can reach tens of degrees or more, raising requirements on the temperature stability for wearable devices. Therefore, conducting an in-depth study of the fundamentals of flexible mechanical force sensors is beneficial for customizing health monitoring systems with desired performance metrics.

### 2.1. Classification of Mechanical Force Sensors

**Figure 3:**
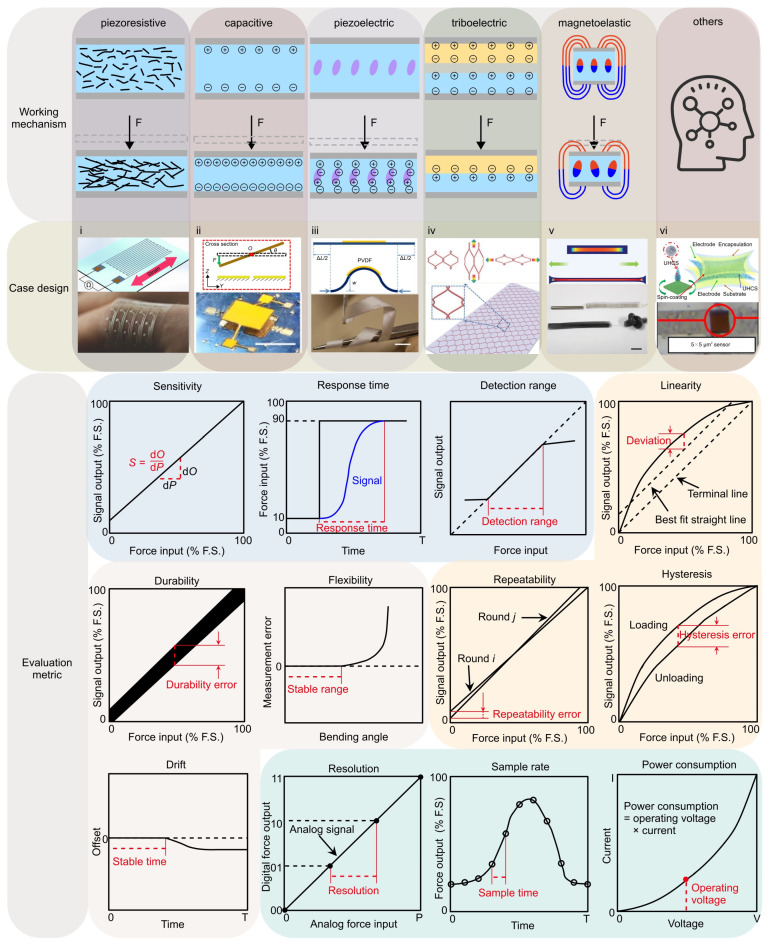
An overview of typical mechanical force sensors classified based on their working mechanisms and key performance metrics. The working mechanisms include piezoresistive effect, capacitive effect, piezoelectric effect, triboelectric effect, magnetoelastic effect and other physics phenomenon (e.g., Fowler–Nordheim tunnelling effect). The twelve commonly used metrics for evaluating force sensors, such as sensitivity, linearity, etc., can be categorized into four major groups, marked by different background colors. These four groups of metrics characterize the sensor’s responsiveness (light blue), measurement accuracy (light orange), operational stability (light pink), and system-level performance (light cyan) when integrated with a signal acquisition board, respectively. Panel (**i**) is adapted with permission from Ref. [123]. Copyright 2020, Wiley-VCH. Panel (**ii**) is adapted with permission from Ref. [75]. Copyright 2021, Springer Nature. Panel (**iii**) is adapted with permission from Ref. [163]. Copyright 2013, Springer Nature. Panel (**iv**) is adapted with permission from Ref. [164]. Copyright 2018, Wiley-VCH. Panel (**v**) is adapted with permission from Ref. [165]. Copyright 2022, Wiley-VCH. Panel (**vi**) is adapted with permission from Ref. [166]. Copyright 2020, Springer Nature.

According to their different working mechanisms, the flexible mechanical force sensors can be classified into six categories (i.e., piezoresistive, capacitive, piezoelectric, triboelectric, magnetoelastic, and other force sensors) (Figure 3). Here, we introduce commonly used component materials, and provide illustrative design cases for each type of working mechanism.

(1) The piezoresistive effect is a phenomenon in which the electrical resistance of a material changes in response to applied mechanical force, originating from alterations in the material’s crystal structure when subjected to mechanical deformations. Due to their simple design and ease of read-out, piezoresistive force sensors were widely investigated. Typically, metals and semiconductors are used as the sensing material, while polymers serve as the soft substrate. For example, Valentine et al. fabricated a biocompatible and highly stretchable force sensor through hybrid 3D printing [123]. The developed Ag thermoplastic polyurethane (TPU) inks could produce mechanically robust, stretchable conductors, resulting in a consistent and repeatable electrical response in the fabricated force sensor.

(2) The capacitive force sensor relies on changes in capacitance to achieve measurements of mechanical force. This type of force sensor typically consists of two conductive plates separated by a dielectric material. Usually, conductive materials like metal, carbon nanotubes, graphene, or conductive polymers can be exploited as the electrodes, while air gaps or non-conductive polymers serve as the dielectric material. For example, Ye and Zhang et al. utilized controlled compressive buckling assembly strategy to fabricate a 3D seesaw-like capacitive sensor with a tunable sensitivity [75].
(3) The piezoelectric effect is an interesting phenomenon observed in specific materials that can produce an electric charge when subjected to mechanical loadings. This usually occurs as a result of the reconfiguration of charged atoms or molecules within the material. Commonly used piezoelectric materials include lead zirconate titanate (PZT) ceramics, lead magnesium niobate-lead titanate (PMN-PT) ceramics, and polyvinylidene fluoride (PVDF). For instance, Persano et al. developed a large-area, flexible piezoelectric material comprising sheets of electrospun fibers made from the polymer poly(vinylidenefluoride-co-trifluoroethylene) (P(VDF-TrFe)) [163]. The force sensor based on such fiber arrays exhibits an ultra-high sensitivity, allowing measurement of very low pressures (e.g., 0.1 Pa).
(4) The triboelectric effect is a phenomenon where specific materials become electrically charged upon contact and subsequent separation. To develop triboelectric force sensors, the researchers usually use copper, silver, carbon, polymer hybrid electrode materials, and flexible ion-gel electrode materials as the electrode materials. For example, Dong et al. reported a stretchable and washable skin-inspired triboelectric nanogenerator through embedding the planar and designable conductive yarn network into the flexible elastomer [164].
(5) The magnetoelastic effect is a phenomenon in which the mechanical properties of a material change in response to an applied magnetic field. Typically, magnetoelastic materials include ferromagnetic materials (e.g., iron, nickel, and cobalt-based alloys), ferrimagnetic materials (e.g., magnetite and yttrium iron garnet) and soft magnetostrictive composites (e.g., blended NdFeB-Ecoflex). For example, Zhang et al. developed an innovative force sensor based on a flexible NdFeB magnet. This sensor exhibits a high stretchability (>150%), a rapid response time (~30 milliseconds), and an outstanding linearity (R^2^ > 0.98) [165].
(6) Other physics phenomenon adopted in designing force sensors includes the Fowler–Nordheim tunnelling effect, optical grating, resonant circuit, among others. Such unusual sensing mechanisms can offer excellent sensing performance in certain aspects of mechanical force sensors. For example, the Fowler–Nordheim tunneling effect is a quantum mechanical phenomenon in which electrons tunnel through a potential energy barrier when exposed to a strong electric field. Based on this mechanism, Shi et al. fabricated a flexible pressure sensor with ultrahigh sensitivity (260.3 kPa^−1^ at 1 Pa), sensing density, and transparency [166].

### 2.2. Evaluation Metrics of Force Sensors

To provide a comprehensive assessment of the performance of a force sensor, many different types of device tests are required, from which evaluation metrics can be obtained. In this review, we list twelve key evaluation metrics of force sensors (Figure 3), including sensitivity, response time, detection range, linearity, hysteresis, repeatability, flexibility, durability, drift, resolution, sample rate, and power consumption. These evaluation metrics can be concluded into four categories (Figure 3), characterizing the sensor’s responsiveness, measurement accuracy, operational stability, and system-level evaluation metrics when integrated with a signal acquisition board, respectively. For clarity, we introduce the definition of the twelve metrics in this section.

(1) Sensitivity is defined as the change of the observed physical quantity given a unit change of the force. The specific expression for the force sensor can be written as:

(1) $$S=\frac{dQ}{dP},\;$$

where *S* represents the sensitivity, while *Q* and *P* stand for the quantitative output signal and applied force, respectively. A high sensitivity enables the sensor to detect small force changes and generate a correspondingly larger output signal, ideal for precise force measurements.

(2) Response time is defined as the time consumption that the sensor needs to apply the changes made in the input force to the output signal. Typically, we use the time it takes for the sensor to rise from 10% to 90% of its full-scale range as the response time. The specific expression is given by:

(2) $$T_{\mathrm{response}}=t{│}_{P=10\%P_{\max}}^{P\;=90\%P_{\max}},$$

where $P_{\max}$ denotes the maximum detection range of the sensor. A short response time means that the force sensor can quickly detect and react to changes in applied force, providing a rapid response of its output signal to reflect those changes.

(3) Detection range refers to the span of force within which the force sensor can provide precise and meaningful force measurements, as written by:

(3) $$\left[P_{\min},\;P_{\max}\right],\;$$

where $P_{\min}$ and $P_{\max}$ denote the lower and upper bound of the detection range. A wide detection range means that the force sensor exhibits robust performance characteristics (e.g., high sensitivity and excellent linearity) across a broad range of force levels. It’s important to note that the specific values of the acceptable evaluation metrics usually depend on the particular application scenarios.

(4) Linearity is defined as the straightness of the output at various equally spaced force points during a loading process, and the error δ

can be used to measure the quality of linearity, as given by:

(4) $$\delta =\mathrm{MAX}(y_{\mathrm{out}}-y_{0}),\;\mathrm{or}\;$$

(5) $$\delta =\mathrm{RMS}(y_{\mathrm{out}}-y_{0}),$$

where $y_{\mathrm{out}}$ represents the output signal and $y_{0}$ represents the output along the best fit straight line. The terms MAX and RMS indicate the operations of finding the maximum value and calculating the root mean square, respectively. A better linearity implies that the measurements are more accurate and reliable.

(5) Hysteresis is defined as the maximum difference in output at any measurement value within the sensor’s specified range, when approaching the point first with increasing and then with decreasing the applied force. The specific expression can be written as:

(6) $$\delta =\mathrm{MAX}(y_{\mathrm{out}-\mathrm{loading}}-y_{\mathrm{out}-\mathrm{unloading}}),\;$$

where $y_{\mathrm{out}-\mathrm{loading}}$ and $y_{\mathrm{out}-\mathrm{unloading}}$ represent the output signal during loading and unloading. A low hysteresis ensures consistent and reliable measurements from the force sensor, regardless of the loading conditions, e.g., whether the unloading is involved in the testing.

(6) Repeatability refers to the consistency of the sensor’s readings when the same magnitude of force is applied multiple times. The difference in the output signals between two measurements can be utilized to quantify the repeatability of the force sensor, i.e.,

(7) $$\delta \;=\mathrm{MAX}(y_{\mathrm{out}-i\mathrm{th}}-y_{\mathrm{out}-j\mathrm{th}}),\;$$

where $y_{\mathrm{out}-i\mathrm{th}}$ and $y_{\mathrm{out}-j\mathrm{th}}$ are the results of the *i*-th and *j*-th measurements, respectively. A good repeatability indicates that the sensor’s readings are highly reproducible and stable during different separate testing.

(7) Flexibility usually refers to the capability of enduring large degrees of bending and twisting deformations, without evident changes in the sensor performance. Typically, the measurement error under different bending (or twisting) angles (*α*) could be used to characterize the sensor’s flexibility, as follows:

(8) $$\alpha_{\max}=\mathrm{MAX}\;\{\alpha \;|\;\delta (\alpha )\;<\;\delta_{\mathrm{threshold}}\},\;$$

where $\delta_{\mathrm{threshold}}$ denotes the maximum acceptable measurement error, determined by the application scenario. A higher flexibility indicates that the sensor could work properly under larger bending/twisting deformations.

(8) Durability in the context of mechanical force sensors denotes their capability of enduring repeated or extended exposure to forces without experiencing notable degradation or damage. Typically, the durability of sensors encompasses two aspects: (1) the stability of the sensor itself under constant environmental influences, and (2) the stability of the evaluation metrics under various environments. The former is mainly determined by the oxidation/corrosion resistance (without force loading) or fatigue property (with force loading) of the component material, over a long period (e.g., months or even years). The latter is usually a short process (several minutes or hours) and contingent upon the service environment (e.g., temperature, humidity, and mechanical stress), because environmental factors highly impact the accuracy and reliability of force measurements. A durable force sensor should display minimal degradation in its measurement capabilities even after undergoing prolonged cyclic loading or rapid environmental changes.
(9) Drift is characterized by the gradual degradation of the sensor, leading to deviations from its originally calibrated state. The drift of force sensor is typically induced by temperature/humidity variations, the aging of sensor components, or other environmental conditions.
(10) Resolution refers to the smallest force increase that the sensor can measure. Typically, signal acquisition boards are utilized to collect analog signals from the force sensors and convert them into digital signals. A higher resolution means that the force sensing system can detect more subtle force variations.
(11) Sample rate, another system-level performance metric, refers to the rate at which the data is read and displayed. A high sample rate can improve the measurement accuracy of the force sensing system, especially for the measurement of dynamic forces.
(12) Power consumption refers to the amount of energy used per unit time, and is usually defined by the product of operating voltage and current [167].

In addition to the twelve key evaluation parameters mentioned above, the overall dimension and weight of these sensors are also important parameters that could affect the suitability for specific applications. Different application scenarios have varying requirements for these two parameters. For example, sensors with reduced weight are highly preferred in aerospace applications.

## 3. Structures and Applications for Different Types of Force Sensors

In this section, we introduce the structure designs and the corresponding medical applications of each type of flexible mechanical force sensor.

### 3.1. Piezoresistive Force Sensors

**Figure 4:**
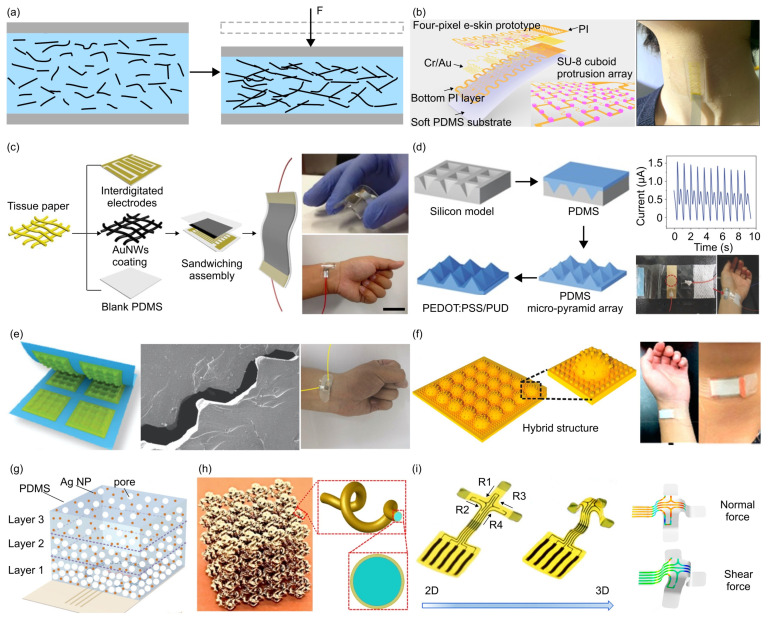
Piezoresistive force sensors. (**a**) Schematic illustration of the piezoresistive effect, which converts the change of force applied to the sensor into the resistance change. (**b**) Schematic of a multifunctional electronic skin (e-skin) based on piezoresistive force sensor. The sensor relies on patterned metal film to offer a broad linear response range. The e-skin can be mounted on the neck to monitor throat movement during the swallowing, which is useful for evaluating swallowing function and the effectiveness of rehabilitation treatment. Adapted with permission from Ref. [78]. Copyright 2021, American Association for the Advancement of Science. (**c**) Schematic of a sandwiched force sensor based on the ultrathin AuNWs-impregnated tissue paper and two thin PDMS sheets. The skin-attachable pressure sensor shows a high sensitivity, and can accurately read out the heartbeat. Adapted with permission from Ref. [172]. Copyright 2014, Springer Nature. (**d**) Schematic of a stretchable piezoresistive force sensor that utilizes micro-pyramid arrays made of PDMS. The sensor could be positioned above the radial artery to monitor the blood pressure in a non-invasive manner. Adapted with permission from Ref. [168]. Copyright 2014, Wiley-VCH. (**e**) Schematic of a flexible microstructured pressure sensor based on the interlocking of dome-topped pyramids. The right panel shows its application for measuring the time-dynamic force induced by the heartbeat. Adapted with permission from Ref. [143]. Copyright 2020, Wiley-VCH. (**f**) Schematic of a piezoresistive force sensor based on a hybrid architecture coated with silver nanowires (AgNWs), featuring both mesoscaled dome and microscaled pillar arrays. The showcased hybrid structure displays a significantly enhanced sensing capability (i.e., sensitivity and detection range) compared to the traditional dome-based counterpart (without pillar arrays), thus enabling accurate monitoring of various types of physical signals. Adapted with permission from Ref. [144]. Copyright 2019, American Chemical Society. (**g**) Schematic of thermosensation-based force sensor that exploits a multilayered porous PDMS/silver nanoparticle (AgNP) sponge with a graded porosity. The pressure sensor possesses both low detection limit and wide measurement range, meeting diverse demands in practical applications. Adapted with permission from Ref. [169]. Copyright 2019, Wiley-VCH. (**h**) Schematic of flexible force sensors based on 3D soft network materials. The developed 3D network materials are defect-insensitive and exhibit mechanical responses similar to those of biological tissues, indicating potential applications in flexible bio-integrated devices. Adapted with permission from Ref. [74]. Copyright 2020, Springer Nature. (**i**) Optical images of the 3D mechanical force sensor in response to normal force, shear force and bending force. Adapted with permission from Ref. [177]. Copyright 2019, American Chemical Society.

Figure 4 illustrates typical structural design strategies for piezoresistive force sensors [55,57,59,62,63,64,74,78,123,143,144,145,155,156,157,168,169,170,171,172,173,174,175,176,177,178,179,180,181,182,183,184,185,186,187,188,189,190,191,192,193,194,195]. Generally, the researchers rely on the measured resistance change of the sensor architecture to determine the applied pressure/force (Figure 4a). For example, Cai et al. attached a patterned metal film (PMF) encapsulated with top and bottom polyimide (PI) onto a soft substrate to create a novel type of electronic skin (Figure 4b) [78]. An array of protrusions is arranged regularly on top of the PMF, such that the PMF between adjacent protrusions can be considered as straight conducting wires. When an external pressure is applied to the top of the e-skin, the rigid protrusions move downward, compressing the soft substrate and creating an arc shape between two protrusions. Hence, the negligible out-of-plane compression of the PMF, caused by the applied pressure, converts into significant bending deformations, resulting in a measurable change in resistance that corresponds to the pressure magnitude. The use of protrusions enhances the pressure sensor’s sensitivity by over 177 times, while maintaining a broad linear (i.e., coefficient of determination (*R*^2^) = 0.995) response range of 80 kPa, as compared to a sensor without protrusions. The developed e-skin also has excellent mechanical flexibility, and can be mounted on skin to monitor the artery pulse and swallowing process. Without using patterned conductive films, Gong et al. developed an efficient, low-cost fabrication strategy to construct a highly sensitive, flexible pressure sensor (Figure 4c) through depositing gold nanowire (AuNWs) into the Kimberly Clark tissue paper [172]. The utilized AuNWs are extremely thin (just around 2 nm), yet span tens of micrometers in length. The slender structure ensures that the AuNWs are mechanically robust and flexible, allowing them to form curved structures without breaking. Gong et al. sandwiched the AuNWs-impregnated tissue paper between two polydimethylsiloxane (PDMS) sheets to form a highly sensitive pressure sensor (sensitivity ~1.14 kPa^−1^). Under external pressure, slight compression of the tissue paper is induced, leading to increased contact between AuNWs and finger electrodes, thereby creating more conductive pathways (Figure 4c). As the tissue paper primarily undergoes elastic deformations, the sensor exhibits minimal hysteresis at 600 Pa and 1 Hz, with a rapid response time (0.05 s) at 5.5 Hz under dynamic loading. This superior performance allows the sensor to detect wrist pulses accurately, under both normal conditions (~66 beats per minute) and after physical exercise (~88 beats per minute).

Additionally, 3D micropatterned structures are widely adopted in the design of flexible mechanical force sensors [59,63,64,74,144,168,169,170,174,176]. Commonly employed geometric configurations of microstructures encompass pyramids, pillars, fibers, and spheres. These elements can be organized into regular arrays of varying sizes and distributions within the layer. Choong et al. coated a blend of conductive polymer—poly(3,4-ethylenedioxythiophene–poly(styrenesulfonate) (PEDOT:PSS) and an aqueous polyurethane dispersion (PUD) elastomer on PDMS micro-pyramid arrays (Figure 4d) [168]. A flat counter electrode with high conductivity is placed on the top of micro-pyramids. Under low pressure, the counter electrode only touches the pyramid tip, creating a high resistance. As the pressure rises, the pyramid spreads, widening the electrode interface and thickening the current path, increasing the current conduction. The sensors could maintain high sensitivity (10.3 kPa^−1^) when stretched by 40%, due to this soft micro-pyramid-structured substrate. Additionally, the excellent sensitivity at low pressures (23 Pa) and fast response time (0.2 s) enable the sensor to be capable of non-invasively measuring the primary features (e.g., distinct systolic and diastolic peaks) of a human pulse waveform. Inspired by wing-locking device of beetles, Pang et al. developed a layered strain-gauge sensor based on nanoscale mechanical interlocking between metal-coated, high-aspect-ratio (high-AR) nanofibers [59]. The interlocking mechanism among neighboring nanofibers, driven by the interplay of attractive van der Waals forces and the deflection force of the nanofibers, renders the sensor’s skin-like capability of detecting various mechanical loads (pressure, shear, and torsion). Similarly, Li et al. devised a piezoresistive force sensor by stacking two flexible PDMS films with a pyramid-wall-grid microstructure, placing them face to face (Figure 4e) [143]. The device demonstrates an exceptional sensitivity, around 383,665.9 kPa^−1^ in the 0–1.6 kPa range and 269,662.9 kPa^−1^ in the 1.6–6 kPa range, allowing high-precision wrist pulse monitoring.

While various 3D microstructures are employed to enhance sensitivity, response time, and repeatability, further improvements in sensitivity at low pressure and the resolution can be achieved through a hierarchical or hybrid structure. As shown in Figure 4f, Ji et al. designed a piezoresistive pressure sensor based on AgNWs-coated hybrid architecture consisting of mesoscaled dome (diameter of 1000 μm) and microscaled pillar arrays (diameter of 50 μm) [144]. The pressure sensor offers a superior sensitivity of 128.29 kPa^−1^ (0−200 Pa), 1.28 kPa^−1^ (0.2−10 kPa), and 0.26 kPa^−1^ (10−80 kPa), and a resolution of 2.5 Pa. Additionally, the output signal remains very stable after a 10,000-cycle loading and unloading test, demonstrating excellent durability. The sensor can be used to accurately monitor different kinds of force sources, e.g., wrist pulse, voice vibration and finger bending/touching. 3D gradient structure and periodical lattice are other two types of commonly used sensing architectures (Figure 4g,h). For example, Zhao et al. utilized a multilayered porous PDMS/silver nanoparticle (AgNP) sponge with a graded porosity to design a skin-inspired pressure sensor with a high resolution of 4.1 Pa and broad range (~200 kPa) [169]. Yan et al. utilized 3D soft network to develop a bio-integrated flexible force sensor capable of detecting small pressures (<5 kPa), while mimicking the mechanical properties of biological tissues [74,196,197]. Notably, Won et al. constructed a 3D multimode tactile sensor via mechanically guided assembly [177]. In particular, the predesigned 3D mesostructures are fabricated through compressive buckling induced during the release of a biaxially prestretched elastomer substrate [198] (Figure 4i). The piezoresistive materials (e.g., monocrystalline silicon nanomembranes) are distributed on the four supporting legs of cross-shaped structures. The force sensor demonstrates mechanical robustness and excellent durability, exhibiting consistent response without variations over 1000 cycles under normal pressures of 5, 10, and 30 kPa. This technology enables separate, simultaneous measurements of multiple static/dynamic mechanical stimuli (e.g., normal force, shear force, and bending) within millisecond, showing promising applications in health monitors, biomedical devices, and human–robotic devices. To extend the battery life of wearable electronics, reduce heat generation in sensors, and promote environmental friendliness, the energy consumption of flexible mechanical force sensors should be kept as low as possible. For example, Qin et al. utilized highly conductive MXenes and the porous structure of polyvinyl butyral to design a flexible piezoresistive sensor with low power consumption (approximately 3.6 × 10^−10^ W), ensuring long-term detection of a series of human activities [199].

### 3.2. Capacitive Force Sensors

**Figure 5:**
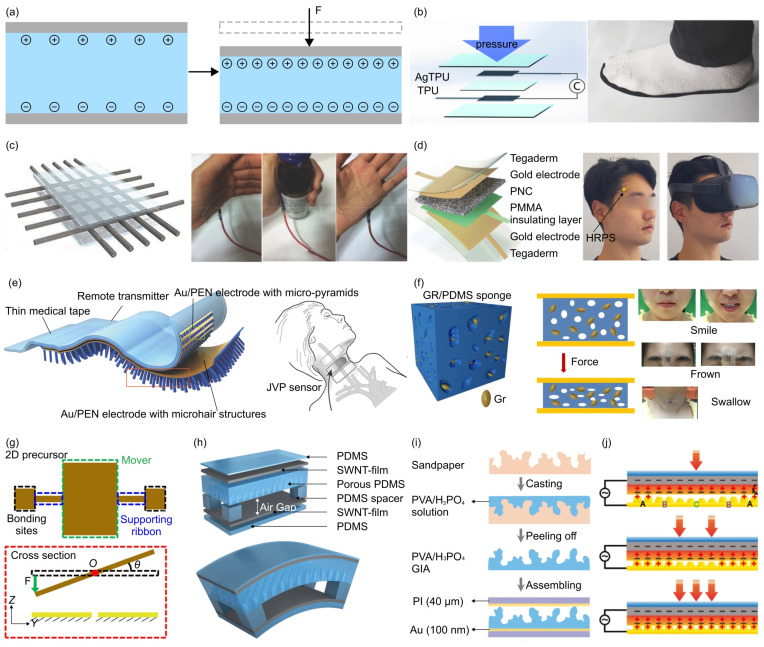
Capacitive force sensors. (**a**) Schematic illustration of the capacitive effect, which converts the force change into the capacitance change. (**b**) Schematic of a capacitive force sensor fabricated through direct ink writing of conductive and dielectric elastomeric materials. The right panel illustrates its application in measurement of the foot pressure distribution through the plantar sensor array. Adapted with permission from Ref. [123]. Copyright 2017, Wiley-VCH. (**c**) Schematic of highly sensitive, wearable, and multimodal all-carbon skin sensors using hierarchically engineered CNT fabrics. The sensor can be attached to the palm to detect human motion. Adapted with permission from Ref. [204]. Copyright 2015, Wiley-VCH. (**d**) Schematic of a flexible hybrid-response force sensor (HRPS) composed of an electrically conductive porous nanocomposite (PNC). The right panel shows it application in measuring subtle pressures from temporal arterial pulse. Adapted with permission from Ref. [203]. Copyright 2015, Wiley-VCH. (**e**) Schematic of highly flexible force sensors with PDMS microhair-structured interfacial layers. This portable sensor, equipped with a wireless transmitter, can measure faint signals originated from the deep-seated internal jugular venous pulses. Adapted with permission from Ref. [112]. Copyright 2015, Wiley-VCH. (**f**) Schematic of a flexible wireless force sensor that uses a graphene/PDMS (GR/PDMS) sponge as the dielectric layer. The sensor can be used to detect finger bending and facial muscle movements due to smile and frown. Adapted with permission from Ref. [205]. Copyright 2019, Springer Nature. (**g**) Schematic of 3D capacitive sensor based on the seesaw-like mesostructure assembled through compressive buckling. Adapted with permission from Ref. [75]. Copyright 2021, Springer Nature. (**h**) Schematic of a stretchable energy harvesting e-skin (EHES) with thin films of SWNTs as the top/bottom electrodes, and PDMS structure and air gap as the dielectric layers. Adapted with permission from Ref. [206]. Copyright 2014, Wiley-VCH. (**i**) Schematic illustration of the fabrication process for the iontronic flexible force sensor consisting of a graded intrafillable architecture (GIA). Adapted with permission from Ref. [200]. Copyright 2020, Springer Nature. (**j**) A capacitive force sensor designed to sandwich the iontronic dielectric layer between a bottom structured electrode and a top polyethylene terephthalate (PET)/indium tin oxide (ITO) electrode. Adapted with permission from Ref. [208]. Copyright 2023, Springer Nature.

Figure 5 illustrates several representative structural design strategies for capacitive force sensors [61,75,110,111,112,120,123,132,147,148,149,150,200,201,202,203,204,205,206,207,208,209,210,211,212,213,214,215,216,217,218,219,220,221,222,223,224,225]. Typically, capacitance force sensors consist of two electrodes and an intermediate dielectric material. Researchers focus on amplifying the capacitance changes caused by sensor deformation through the structural design of the electrode and dielectric (Figure 5a). For example, Valentine et al. created a parallel-plate capacitive sensor using a multilayered architecture by employing hybrid 3D printing (Figure 5b) [123]. This sensor features a deformable dielectric layer made of pure TPU, sandwiched between highly conductive Ag/TPU electrodes both above and below. When external pressure is applied, the thickness of the insulating layer between the two printed electrodes decreases, resulting in a noticeable increase in capacitance. The capacitive sensor demonstrates consistent performances over numerous cycles of applied pressure, and maintains a good linearity (i.e., coefficient of determination (*R*^2^) = 0.996) up to 3 MPa. The hybrid 3D printing technology makes the manufacturing highly efficient, facilitating the fabrication of a plantar sensor array for monitoring trait status. In addition to conductive thin films, networks of conductive wires can also be used as electrodes of capacitive force sensors. Kim et al. utilized hierarchically engineered elastic carbon nanotube (CNT) fabrics to achieve highly sensitive, wearable, and multimodal pressure sensors (Figure 5c) [204]. The sensor exhibits mechanical robustness under various mechanical deformations (e.g., folding, twisting, and stretching conditions), and can be attached to the palm to detect human motion. Beyond a purely CNT fabric-based electrode design, Ha et al. developed an electrically conductive porous nanocomposite (PNC) fabricated by using CNTs-doped Ecoflex (Figure 5d) [203]. PNC serves as the electrode and exhibits hybrid piezoresistive and piezocapacitive responses. The sensitivity of the resulting pressure sensor is significantly enhanced over a wide range of pressures, with values of 3.13 kPa^−1^ within 0–1 kPa, 1.65 kPa^−1^ within 1–5 kPa, 1.16 kPa^−1^ within 5–10 kPa, 0.68 kPa^−1^ within 10–30 kPa, and 0.43 kPa^−1^ within 30–50 kPa. Such enhancement facilitates the measurement of subtle pulsations at the frontal temporal artery and the carotid artery, even under a high preload applied by a virtual reality (VR) headset.

Altering the structural configuration of the dielectric materials is another commonly used design strategy for capacitive force sensors. For example, Pang et al. utilized a pyramid-shaped PDMS (6 μm in width and 3 μm in height) as the dielectric layer and microhair structures (e.g., pillars) as the interfacial layer (Figure 5e) [112]. The stacked layers of the developed pressure sensor consist of polyethylene naphthalene (PEN)/Cr/Au/polyvinyl alcohol (PVA)/PDMS/Au/Cr/PEN/PVA/microhair, resulting in an evident improvement (by approximately 12 times) of the signal-to-noise ratio of the measured radial artery pulse wave. In Figure 5f, Kou et al. employed a graphene/PDMS sponge as the dielectric layer to design a flexible pressure sensor with a broad operation range (0–500 kPa), quick response time (~7 ms), and high resolution (5 Pa) [205]. The sensor is highly sensitive to finger bending and facial muscle movements. Figure 5g–j demonstrate four additional representative designs of capacitive force sensors with structured dielectric layers. An adjustable gap formed through compressive buckling was utilized to design seesaw-like 3D capacitive force sensor with tunable sensitivity (~33 times) and high resolution (~5.22 nN) (Figure 5g) [75]. Figure 5h shows a type of capacitive force sensors with porous PDMS and air gap as the dielectric layers, and single walled nanotubes (SWNTs) films as the top and bottom electrodes [206]. The hybrid dielectric layer endows the force sensor with a high sensitivity (up to ~1.5 kPa^−1^). In addition to dielectric layers with regular/periodic structures (e.g., film, cuboid, pyramids array), Bai et al. utilized a graded intrafillable architecture (GIA) consisting of flexible protruding microstructures to boost the sensitivity (>220 kPa^−1^), the detection range (0.08 Pa–360 kPa), and the resolution (0.0056%) over the full pressure range, simultaneously (Figure 5i) [200]. To further improve the linearity of the capacitive force sensor, the gradient micro-pyramids and ultrathin ionic layer were introduced in the design of sensor architecture (Figure 5j) [208]. The optimized sensor achieves a sensitivity of 33.7 kPa^−1^ within a linear range of 1700 kPa, and a pressure resolution of 0.00725%. This flexible iontronic capacitive pressure sensor can be attached to unconventional body locations to monitor physiological signals, such as detecting tiny pressures (e.g., a weak pulse signal of ~10 Pa) and subtle motions.

### 3.3. Piezoelectric and Triboelectric Force Sensors

**Figure 6:**
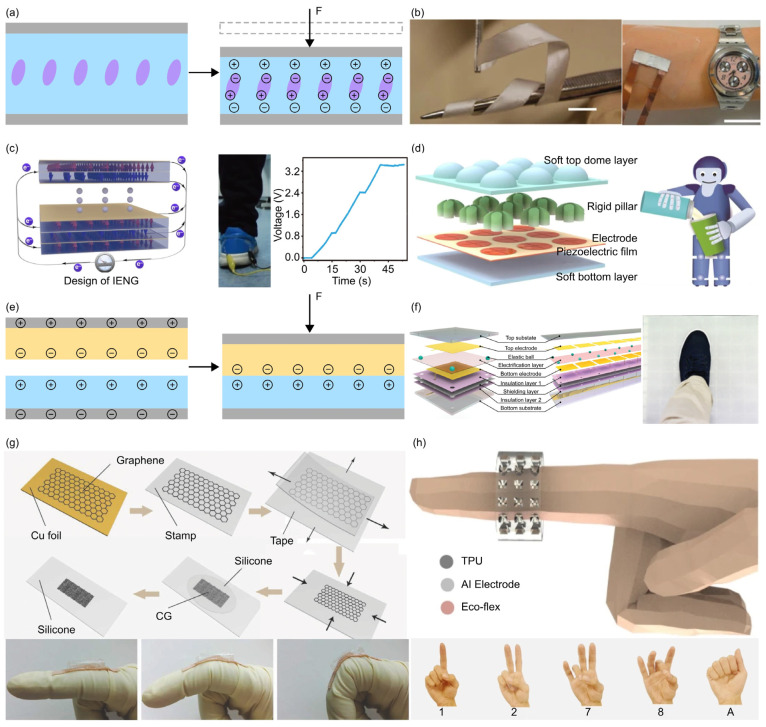
Piezoelectric and triboelectric force sensors. (**a**) Schematic illustration of the piezoelectric effect, which mainly converts the force change into the voltage change. (**b**) Optical image of a piezoelectric force sensor based on highly aligned piezoelectric nanofibers of P(VDF-TrFe). The right panel shows its application on the detection of motion direction when the flexible sensor is mounted on the arm. Adapted with permission from Ref. [163]. Copyright 2013, Springer Nature. (**c**) Schematic of piezoelectric force sensor based on a 3D intercalation electrode with enhanced current density. Adapted with permission from Ref. [227]. Copyright 2020, Springer Nature. (**d**) Schematic of a finger-inspired piezoelectric force sensor with rigid-soft hybrid force-transmission layer. The right panel demonstrates its application in hand grasps, such as holding a plastic bottle into which water drops are added. Adapted with permission from Ref. [76]. Copyright 2022, Springer Nature. (**e**) Schematic illustration of the triboelectric effect, which converts the force change into the charge change. (**f**) Schematic of a self-powered integrated triboelectric sensor array (ITSA). Integrated with a signal processing circuit, ITSA can achieve pressure detection, position identification, and trajectory tracking. Adapted with permission from Ref. [162]. Copyright 2020, Wiley-VCH. (**g**) Schematic of a triboelectric nanogenerator (i.e., TENG) based on crumpled graphene (CG) layers and silicone films. The bottom panel shows its application in body motion monitoring. Adapted with permission from Ref. [244]. Copyright 2017, Wiley-VCH. (**h**) Schematic of TENG force sensor with pyramid-structured silicone rubber film and TPU ring. The bottom panel shows its application in sign language gestures recognition. The characters “1”, “2”, “7”, “8” and “A” denote gesture meanings. Adapted with permission from Ref. [77]. Copyright 2022, Springer Nature.

Figure 6a–d illustrates the working mechanism of piezoelectric force sensors [76,122,125,151,152,163,182,226,227,228,229,230,231,232,233,234,235,236,237,238,239] and introduces several representative structural design strategies (e.g., fiber arrays, stacked layers, rigid-soft hybrid structure) of sensor architecture. Specifically, Persano et al. utilized high-density arrays of aligned piezoelectric nanofibers (e.g., P(VDF-TrFe)) to develop a piezoelectric force sensor with large area (tens of cm^2^) (Figure 6b) [163]. The resulting force sensors, capable of responding to both compressive and bending forces, are mechanically robust and highly sensitive (~0.4 mV Pa^−1^) in the low-pressure regime (0.1 Pa), making them suitable for applications in detecting human motions. Gu et al. introduced boundary interfaces into piezoelectric force sensors by stacking multiple piezoelectric layers, each sandwiched between a pair of electrodes (Figure 6c) [227]. These boundary interfaces increase the quantity of surface polarization charges, thereby enhancing the current density. The voltage and current signals can reach approximately 10 V and 1 μA, when the sensor is subjected to a compression of 12 kPa, making it applicable as a human-motion-charged power source. To enhance the sensitivity of piezoelectric force sensors at high-frequency dynamic stimuli, Zhang et al. designed a rigid-soft hybrid force transmission layer composed of soft polymer and rigid pillars (Figure 6d) [76]. Such a rigid-soft hybrid tactile sensor (RSHTS) is fabricated by combining the sensory layer (a piezoelectric film attached with patterned electrodes) with a soft bottom substrate. The RSHTS exhibits a high sensitivity (346.5 pC N^−1^), a wide bandwidth (5–600 Hz), and a broad force detection range (0.009–4.3 N). More importantly, the RSHTS array can detect multiple force directions (i.e., ±X, ±Y, and ±Z), and is capable of recognizing high-frequency vibrations, such as measurements of impact forces and monitoring of water pouring processes.

Recently, the triboelectric effect has found widespread applications in the design of flexible mechanical force sensors [77,153,154,162,164,240,241,242,243,244,245,246,247,248,249,250,251,252,253,254], particularly those with self-powered capabilities. In 2012, Professor Zhonglin Wang first proposed the concept of triboelectric nanogenerator (TENG) [255]. Typically, TENGs have four primary working modes, including vertical contact-separation, lateral sliding, single-electrode, and freestanding triboelectric-layer modes [247]. Triboelectric force sensors primarily function in the vertical contact-separation mode (Figure 6e). For example, Wang et al. developed a large-area, self-powered integrated triboelectric sensor array (ITSA) by combining a triboelectric sensor array and an array of bilateral switches (CD4066) [162]. The architecture of the triboelectric sensor array comprises a top substrate layer (silicone), a top electrode layer (woven conductive fabric), a layer of elastic balls (with a 5 mm diameter), an electrification layer (polytetrafluoroethylene (PTFE) film), a bottom electrode layer (woven conductive fabric), two insulation layers, and a grounded shielding layer (Figure 6f). The large-area flexible pressure sensor is capable of recording the position and contacting contour of the feet during foot stepping experiments. To facilitate the integration of triboelectric force sensors with wearable electronics, many studies focused on reducing the sensor size, while maintaining a high output voltage. For example, Chen et al. developed a stretchable triboelectric nanogenerator (TENG) based on crumpled graphene and demonstrated its application in detecting finger/wrist motions (Figure 6g) [244]. The output voltage varies from 32 to 56 mV, as the bending angle changes from ~30° to ~90°. The sensor could also serve as an energy harvester under compression mode, stretching mode, and their hybridized mode. Typically, triboelectric force sensors consist of layers of dielectric materials and electrodes. Similar to the design strategies exploited in capacitive pressure sensors, 3D architectures can also be leveraged when designing the dielectric layer of a triboelectric force sensor. For example, Sun et al. developed a TENG tactile sensor by combining a layer of silicone rubber film with pyramid structures and a TPU ring (Figure 6h) [77]. When the finger bends, the contact area between the finger skin and the pyramid structures increases because of muscle swelling. This leads to changes in electrical potential at the output electrodes, generated by the triboelectrification occurring on the contact surface. The sensor architecture and operation mode ensure the precise detection of finger bending and gripping. Integrated with the IoT module and the machine learning algorithms, the tactile-perception ring can be further utilized in real-time gesture/sign language recognition.

### 3.4. Mechanical Force Sensors Based on Magnetoelastic Effect and Other Physics Phenomenon

**Figure 7:**
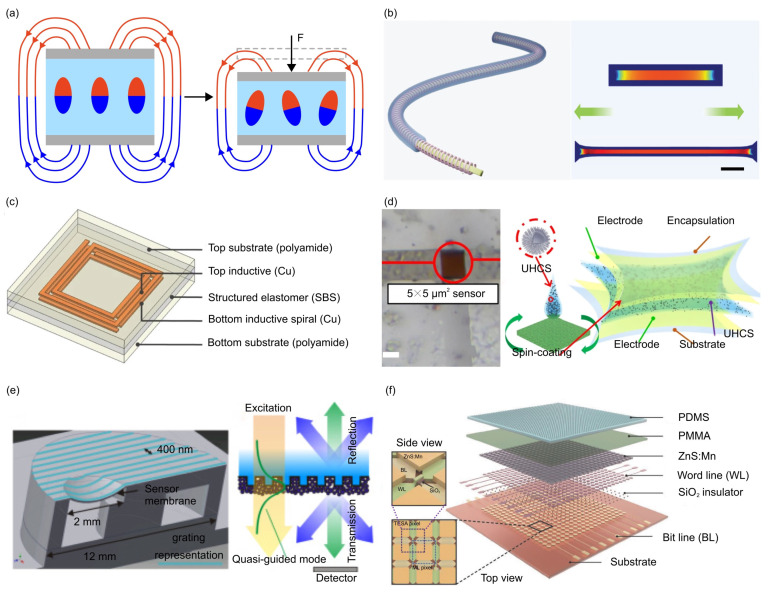
Force sensors based on magnetoelastic effect and other mechanisms. (**a**) Schematic illustration of the magnetoelastic effect, which converts the force change into the magnetic flux density change. (**b**) Schematic of a magnetoelastic force sensor based on a flexible NdFeB magnet, which can convert the mechanical energy associated with finger movement into electrical energy based on Faraday’s law of electromagnetic induction. Adapted with permission from Ref. [165]. Copyright 2022, Wiley-VCH. (**c**) Schematic of pressure monitoring based on passive resonant sensors. Here, a pressure-sensitive capacitive element is integrated with an inductive antenna to form the resonant circuit. Adapted with permission from Ref. [258]. Copyright 2014, Springer Nature. (**d**) Schematic of quantum effect-based flexible and transparent force sensors. Adapted with permission from Ref. [166]. Copyright 2020, Springer Nature. (**e**) A force sensor based on flexible photonic crystal membrane. Adapted with permission from Ref. [259]. Copyright 2015, Optical Society of America. (**f**) A force sensor based on optical and triboelectric dual-mode sensing. Adapted with permission from Ref. [260]. Copyright 2017, Wiley-VCH.

The magnetoelastic effect, a phenomenon in which the mechanical properties of materials change in response to an applied magnetic field (Figure 7a), is typically observed in rigid alloys [60]. Recently, many soft magnetoelastic composites were reported and exploited in the development of mechanical force sensors [60,165,256,257]. For example, Zhang et al. fabricated a flexible NdFeB magnet by blending the magnetic powers with Ecoflex [165], which allows conversion of mechanical deformation energy into electrical energy, in accordance with Faraday’s law of electromagnetic induction. The resulting flexible sensor (Figure 7b) exhibits a high stretchability (>150%), a short response time (30 ms), and an excellent linearity (R^2^ > 0.98), enabling real-time interactive gesture recognition.

Other unconventional physical phenomena, such as resonant circuits, the Fowler–Nordheim tunneling effect, and optical grating, were also harnessed in the design of mechanical force sensors with superior performances [166,258,259,261]. For example, a resonant circuit was formed by integrating pressure-sensitive capacitive element and an inductive antenna (Figure 7c) [258]. The applied force alters the capacitance, subsequently changing the resonant frequency of the circuit. This force sensor surpasses the operating frequency limits of traditional strategies, and exhibits insensitivity to lossy tissue environments. To create a mechanical force sensor capable of producing a substantial electrical signal change in response to minuscule deformations, Shi et al. harnessed the Fowler–Nordheim tunneling effect in the development of a flexible pressure sensor with a multilayer architecture (Figure 7d) [166]. Figure 7e illustrates a flexible mechanical force sensor formed by a periodically nanostructured Bragg grating on a flexible PDMS membrane [259]. The applied force induces deformations in the photonic crystal membrane, resulting in a shift of the guided mode resonances. The change in optical intensity, represented by a shift in green color, can be measured using crossed polarization filters, thereby allowing the determination of the applied force. The estimated resolution of the force sensor is 160 Pa, showing promise for applications as an implantable IOP sensor. Flexible mechanical force sensors based on different operation mechanisms usually show different advantages and shortcomings. Strategic combination of various mechanisms provides a possible route to enhance the sensor’s performance in multiple aspects simultaneously. Wang et al. developed a full dynamic-range pressure sensor based on optical and electrical dual-mode sensing (Figure 7f) [260]. The triboelectric sensor enables highly sensitive pressure sensing in low-pressure regimes (<100 kPa), while the mechanoluminescent sensor performs well in high-pressure regimes (>1 MPa).

## 4. Summary and Perspectives

This review offers a comprehensive overview of recent advancements in the development of flexible mechanical force sensors, along with perspectives on remaining challenges and opportunities in this field. It encompasses both fundamental and applied aspects of the field, such as sensing mechanisms and structural design strategies of force sensors, as well as a wide range of biomedical applications (i.e., healthcare and diagnosis) where these force sensors with tailored architectures have proven to be highly valuable. Although remarkable progress has been achieved as summarized herein, many challenges and open opportunities exist in this exciting area. Flexible mechanical force sensors with novel capabilities, enhanced performances and increased degree of integration are still worthy of future exploration. In particular, development of innovative sensor architecture and intelligent sensing systems is essential for achieving enhanced sensing performance and realizing personalized, long-term healthcare. Besides, the reverse design and on-demand optimization of flexible mechanical force sensors also represent an important yet underexplored direction, because specific application scenarios in healthcare and diagnosis usually pose varying requirements for sensor architecture and performance.

### 4.1. Developing Novel Sensor Architectures

**Figure 8:**
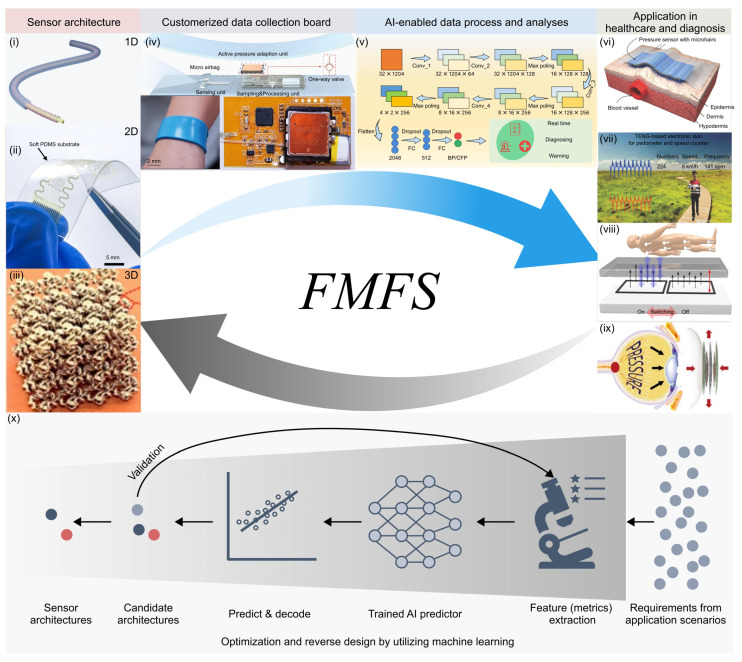
A vision for the problem-driven force sensing system design and optimization. Flexible force sensors can be classified into one-dimensional, two-dimensional, and three-dimensional based on their structural features. Customized data collection boards can collect analog signals from the sensor end, and transmit these signals to the display or mobile end. Compared to traditional algorithms, AI-enabled algorithms can extract vital physiological information from sensory signals with higher accuracy and efficiency. Ultimately, flexible force sensor systems are developed for practical applications, e.g., health monitoring and medical diagnostics. To meet the high demands of the medical instrument market, we need to further customize and optimize the performance of flexible force sensors for specific application scenarios. Development of an AI-enabled reverse design strategy of the sensor architecture could help accelerate the design process. The arrows show the flow of forward and reverse design. Panel (**i**) is adapted with permission from Ref. [165]. Copyright 2022, Wiley-VCH. Panel (**ii**) is adapted with permission from Ref. [78]. Copyright 2021, American Association for the Advancement of Science. Panel (**iii**) is adapted with permission from Ref. [74]. Copyright 2020, Springer Nature. Panel (**iv**) is adapted with permission from Ref. [56]. Copyright 2023, Springer Nature. Panel (**v**) is adapted with permission from Ref. [113]. Copyright 2023, American Association for the Advancement of Science. Panel (**vi**) is adapted with permission from Ref. [112]. Copyright 2015, Wiley-VCH. Panel (**vii**) is adapted with permission from Ref. [164]. Copyright 2018, Wiley-VCH. Panel (**viii**) is adapted with permission from Ref. [57]. Copyright 2021, Springer Nature. Panel (**ix**) is adapted with permission from Ref. [145]. Copyright 2017, Springer Nature. Panel (**x**) is adapted with permission from Ref. [146]. Copyright 2023, Springer Nature.

In general, flexible force sensors can be categorized into three classes (i.e., one-dimensional, two-dimensional, and three-dimensional) based on their structural characteristics (Figure 8). A variety of structural design strategies, such as networks of conductive wires, arrays of nanofibers, periodic placement of pyramids, pillars, or other protrusions, combinations of multiple functional materials, multi-layer film stacking, and hierarchical/gradient architectural designs, are employed to improve one or several performance metrics in sensitivity, response time, linearity, detection range, flexibility, and/or resolution. However, concurrently enhancing multiple performance metrics remains very challenging. Novel architectures integrated with functional materials require further investigation to meet these requirements. More importantly, when deployed in practical applications, flexible mechanical force sensors would encounter environmental challenges like high humidity, high-frequency friction, corrosion, and others. Therefore, improved environmental adaptability should be taken into account in the sensor architecture design. In the future, it is expected that flexible mechanical force sensors will offer not only reliability and stability in use, but also comfort in wearability.

### 4.2. Intelligent Mechanical Force Sensing Systems with Integrated Hardware and Software

To facilitate the integration of force sensors with wearable and portable healthcare and diagnostic electronics, customized data collection boards and data processing algorithms are required (Figure 8). These components collect analog signals from the sensor end, extract physiological information from the collected force signals, and transmit this information to the display or mobile end, allowing for real-time feedback. Such a compact hardware system shows high demands on the architecture and performance of the force sensor, encompassing factors like the sensor’s overall size, power efficiency, and data transmission capabilities. Additionally, the measured force signals typically contain valuable physiological feature information, and is easily susceptible to the interference from sensor deformations and body movements. Artificial intelligence (AI)-enabled algorithms can allow identification of multiple crucial physiological information from sensory data with increased accuracy and efficiency [55]. Despite the continuous expansion of multi-modal data within the biomedical and health area, the emergence of large AI models heralds a new era for health informatics [262].

### 4.3. Problem-Driven Design of Flexible Mechanical Force Sensors

The ultimate objective of the flexible mechanical force sensor discussed here is to detect force signals emanating from human tissues and organs, thereby offering healthcare service and diagnostic solutions for customers and patients (Figure 8). The measurement of various health indicators places distinct requirements on flexible mechanical force sensors, depending on factors such as the shape of the sensing area, the magnitude and frequency of forces generated by physiological activities, and the operational environment of the devices. To meet the increased demands of the medical instrument market, we need to further customize the performance of flexible force sensors for specific application scenarios. Specifically, the requirements stemming from the applications could be translated into the twelve metrics discussed above. These metrics subsequently function as the design objectives for the force sensor. The diverse sensor architectures, in turn, provide the design space, and the implementation of an AI-enabled reverse design strategy for the sensor architecture could help accelerate the process of customization [146,263,264,265,266,267,268,269,270,271,272,273,274,275,276,277,278,279,280].

## Data Availability

The data used to support the findings of this study are available from the corresponding author upon request.
